# Fungal‐host diversity among mycoheterotrophic plants increases proportionally to their fungal‐host overlap

**DOI:** 10.1002/ece3.2974

**Published:** 2017-04-22

**Authors:** Sofia I. F. Gomes, Vincent S. F. T. Merckx, Serguei Saavedra

**Affiliations:** ^1^ Naturalis Biodiversity CenterLeidenThe Netherlands; ^2^ Institute of Environmental SciencesLeiden UniversityLeidenThe Netherlands; ^3^ Department of Civil and Environmental EngineeringMITCambridgeMAUSA

**Keywords:** mycoheterotrophic interactions, mycorrhizal cheaters, niche overlap, niche width, plant coexistence, plant–belowground interactions

## Abstract

The vast majority of plants obtain an important proportion of vital resources from soil through mycorrhizal fungi. Generally, this happens in exchange of photosynthetically fixed carbon, but occasionally the interaction is mycoheterotrophic, and plants obtain carbon from mycorrhizal fungi. This process results in an antagonistic interaction between mycoheterotrophic plants and their fungal hosts. Importantly, the fungal‐host diversity available for plants is restricted as mycoheterotrophic interactions often involve narrow lineages of fungal hosts. Unfortunately, little is known whether fungal‐host diversity may be additionally modulated by plant–plant interactions through shared hosts. Yet, this may have important implications for plant competition and coexistence. Here, we use DNA sequencing data to investigate the interaction patterns between mycoheterotrophic plants and arbuscular mycorrhizal fungi. We find no phylogenetic signal on the number of fungal hosts nor on the fungal hosts shared among mycoheterotrophic plants. However, we observe a potential trend toward increased phylogenetic diversity of fungal hosts among mycoheterotrophic plants with increasing overlap in their fungal hosts. While these patterns remain for groups of plants regardless of location, we do find higher levels of overlap and diversity among plants from the same location. These findings suggest that species coexistence cannot be fully understood without attention to the two sides of ecological interactions.

## 1 Introduction

Mycorrhizal fungi play a crucial role for plant survival (Smith & Read, 2008). In mycorrhizal interactions, mycorrhizal fungi facilitate the uptake of essential resources for plant metabolism, such as water and soil minerals (Raven, Evert, & Eichhorn, 1999). Generally, in exchange, plants transfer photosynthetically fixed carbon to their mycorrhizal partners (Smith & Read, 2008). Occasionally, however, plants do not give back carbon, but instead obtain it from the mycorrhizal fungi as replacement for photosynthesis (Leake, 1994; Merckx, Bidartondo, & Hynson, 2009). This results in an antagonistic interaction between plants and their fungal hosts. Specifically, these interactions are called mycoheterotrophic (MH) interactions and can occur in a single developmental stage (e.g., in orchids, and some ferns and lycopods) or during the entire life cycle of a plant (fully mycoheterotrophic plants) (Merckx & Freudenstein, 2010; Winther & Friedman, 2008). MH interactions represent a nonmutualistic mode of life that occurs in nearly all major lineages of land plants, involving more than 20,000 plant species (Merckx, 2013). In general, the fungal‐host diversity available for these plants is restricted as MH interactions often involve more narrow lineages of mycorrhizal fungi than non‐MH interactions (Bidartondo et al., 2002). Unfortunately, little is known whether fungal‐host diversity may be additionally modulated by plant–plant interactions through shared hosts. Yet, this may have important implications for plant competition and coexistence (Bever et al., 2010).

**Figure 1:**
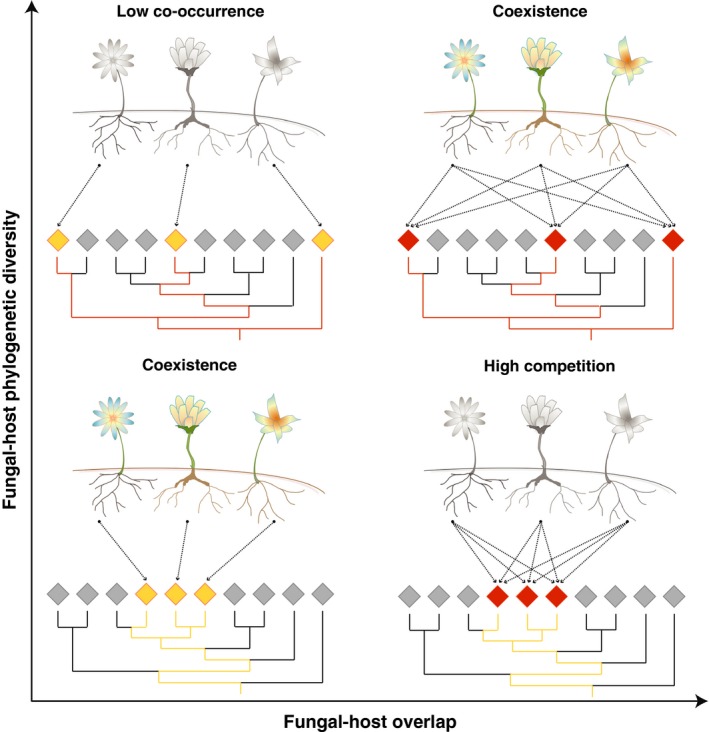
Illustration of possible fungal‐host patterns among mycoheterotrophic plants. On the vertical and horizontal axes, the figure illustrates, respectively, an increase in fungal‐host diversity and fungal‐host overlap among MH plants. The bottom right panel represents a scenario for plants with high chances of competitive exclusion given by their large fungal‐host overlap and their small fungal‐host diversity (using similar functional traits). The top left panel represents a scenario for plants with low chances of co‐occurring in the same space given by their small fungal‐host overlap and their large fungal‐host diversity (using different functional traits), which could be difficult to find in a common place. The diagonal panels then represent the scenarios for plants with a higher chance of coexistence given by their symmetry between fungal‐host overlap and fungal‐host diversity, which could lead to maximize co‐occurrence (exploit available resources) and to minimize competitive exclusion

Recent studies have shown that the diversity of mycorrhizal fungi is strongly associated with plant community composition (Davison, Öpik, Daniell, Moora, & Zobel, 2011; Martínez‐García, Richardson, Tylianakis, Peltzer, & Dickie, 2015; Peay, Baraloto, & Fine, 2013) and habitat conditions (Hazard et al., 2013). For instance, in the case of MH interactions, a given group of plant species can be exploiting either closely or distantly related fungal hosts (see Figure 1). Additionally, this same group of plants can have either a weak or a strong fungal‐host overlap (see Figure 1). The combination of these two factors depends on plant niche and have been shown to be determinant for plant coexistence (Levine & HilleRisLambers, 2009; Levins, 1968; Rohr et al., 2016). According to niche theory (Loreau, 2010; MacArthur & Levins, 1967), species coexistence is a function of their their niche width and niche overlap (Chesson, 2000). Competitive exclusion among species is high when their potential niche overlap is large and their combined niche width is small. Similarly, the chances of co‐occurrence among species in the same niche space is low when their potential niche overlap is small and their combined niche width is large. Species coexistence (co‐occurrence and no exclusion) then is expected to happen when niche overlap and niche width are symmetric (Chesson, 2000; Tilman, 2011) (see Figure 1—diagonal). Niche delimitation is never straightforward due to our often lack of a priori knowledge about the resources and functional traits defining the niche dimensions of a species (Kraft, Godoy, & Levine, 2015). Defining the niche of fungal hosts of mycoheterotrophic plants is as challenging as for other groups of organisms, but one potential hypothesis is that the higher the fungal‐host diversity of mycoheterotrophic plants, the broader their niche. Thus, species coexistence may be favored under symmetric patterns of fugal‐host overlap and diversity.

To work on the above hypothesis, we use a system where the mycorrhizal interaction involves mycoheterotrophic plants. In addition, these plants are associated with arbuscular mycorrhizal fungi (phylum Glomeromycota), which are associated with more than 80% of land plants. Therefore, this association represents one of the most ancient and abundant mycorrhizal interaction among plants on a global scale (Smith & Read, 2008; Strullu‐Derrien et al., 2001). Here, we investigate MH interactions by analyzing the observed patterns of associations between MH plants and their fungal hosts in a niche framework. In particular, we study how the phylogenetic diversity of arbuscular mycorrhizal hosts varies among individual MH plants, and how this diversity is modulated and shared among groups of MH plants.

## 2 Methods

### 2.1 Sampling sites and mycoheterotrophic species

The geographic range of MH plants associated with arbuscular mycorrhizal fungi is mostly restricted to tropical rainforests worldwide (Leake, 1994). Neotropical forests harbor the largest species diversity compared to the paleotropical forests. In the neotropics, the two biomes with the highest diversity of MH species are the Amazon forest and the Atlantic forest (Merckx, 2013). We collected MH plants in these two biomes in French Guiana and Brazil, respectively (see Fig. S3). The sampled sites in French Guiana were low land coastal plain forests (Guitet, Brunaux, Granville, Gonzalez, & Richard‐Hansen, 2015), and in Brazil were also low lands in Ombrophilous dense coastal forests (Veloso, Rangel Filho, & Lima, 1991). Due to the ephemeral nature of MH plants, it is only possible to collect them during their flowering period. Most MH species flower after the rainy season, from July until November. All collections were made during this period.

We visited 15 localities, 10 of which in the Amazon forests and five in the Atlantic forests. We considered all the individuals of the same species found within 4 × 4 m to be part of the same population. Populations of MH species were separated from each other with a minimum of 30 m. In each population, we collected at least one individual and a maximum of ten individuals per species. We focused on three of the four MH plant families distributed in the sampled area, namely Burmanniaceae, Gentianaceae, and Triuridaceae. We did not target species of Thismiaceae, the fourth family of MH plants in the area, as all neotropical species are extremely rare. In the 15 localities, we identified 54 populations of MH species. In total, we collected root samples of 140 specimens of 20 MH plant species, covering more than a quarter of the described arbuscular mycorrhizal MH species for South America. See Supporting Information for further details about the sampling.

### 2.2 Fungal‐host diversity in single mycoheterotrophic plants

To study fungal‐host patterns, first we investigated the arbuscular mycorrhizal fungal‐host diversity that can be potentially associated with single MH plants. This information was obtained through DNA sequencing of roots of arbuscular mycorrhizal MH plants. For each of the 140 specimens, immediately after collection, root samples were washed with distilled water and stored in 2% CTAB buffer at $-20^{\circ }C$ until further processing. Subsequently, DNA was extracted using the NucleoSpin Soil kit (Macherey‐Nagel Gmbh and Co., Düren, Germany). Next‐generation DNA sequencing of each root sample was used to identify the arbuscular mycorrhizal hosts that can be potentially associated with each MH plant species. We sequenced the ITS2 region using the primers fITS7 (5′‐GTGARTCATCGAATCTTTG‐3′) (Ihrmark et al., 2012) and ITS4 (5′‐TCCTCCGCTTATTGATATGC‐3′) (White, Bruns, Lee, & Taylor, 1990). In total, we found 138 operational taxonomic units (OTUs) identified as Glomeromycota by querying against UNITE database (version 6.0, 10.09.2014) using the BLAST algorithm. Hereafter, we refer to the fungal OTUs as fungal hosts. See Supporting Information for more details about the sequencing. Raw sequences are deposited in the NCBI Short Read Archive under the project number PRJNA339563.

To generate the phylogenetic tree for each family of MH plant species, we reconstructed the phylogenetic relationships between the species for each family by reanalyzing previously published datasets of Burmanniaceae (Merckx, Huysmans, & Smets, 2010), Triuridaceae (Mennes, Smets, Moses, & Merckx, 2013), and Gentianaceae (Merckx et al., 2013). For Triuridaceae, we included newly sequenced data for *Soridium spruceanum* (GenBank accession number KX756649). We combined the resulting trees based on divergence ages taken from Magallón, Gómez‐Acevedo, Sánchez‐Reyes, and Hernández‐Hernández (2015). Only the 20 taxa from this study were kept in the phylogeny shown in Fig. S2.

To generate the host phylogenetic tree, we used an alignment with the 138 Glomeromycota fungal OTUs with MAFFT 7.017 (Katoh, Misawa, Kuma, & Miyata, 2002) implemented in Geneious Pro 6.1.4 (Biomatters, Auckland, New Zealand). Reference sequences of the accepted genera in the phylum were added as a backbone to the tree to support and better deduce the phylogenetic position of each OTU (Krüger, Krüger, Walker, Stockinger, & Schüßler, 2012; Öpik et al., 2010). We reconstructed a maximum‐likelihood tree using the GTR+I+G substitution model as selected with jModeltest 2.3.1 (Darriba, Taboada, Doallo, & Posada, 2012) under the Akaike information criterion. The resulting highest‐likelihood tree was transformed into an ultra‐metric tree using *compute.brlen* and *vcv* commands in the *R‐ape* package. The phylogeny of the 138 Glomeromycota OTUs is shown in Fig. S3. The alignment and tree topology are archived in the database TreeBASE (http://www.treebase.org; submission ID 20259).

To calculate the effect of phylogenetic relatedness on the number of fungal hosts among MH plants (phylogenetic signal), we computed the Mantel test correlation between the phylogenetic distance matrix between plants and the dissimilarity matrix between the number of fungal hosts per plant. The phylogenetic distances were extracted from the plants phylogenetic tree, and the dissimilarity matrix was calculated by $|d_{i}-d_{j}|$, where $d_{i}$ and $d_{j}$ are the number of fungal hosts associated with plant i and j, respectively (Saavedra, Rohr, Gilarranz, & Bascompte, 2014). Separately, phylogenetic relatedness on the number of fungal hosts was investigated among MH plants species that belong to the same location.

To calculate the phylogenetic signal on the shared fungal hosts among MH plants, we computed the Mantel test correlation between the phylogenetic distance matrix between plants and two dissimilarity matrices between the shared hosts. The phylogenetic distance matrix is the same as above, whereas the dissimilarity matrices here were calculated using two different measures. The Bray–Curtis measure 1 − (2*C*_*ij*_)/(*d*_*i*_ + *d*_*j*_), where $C_{ij}$ is the number of shared hosts between plant i and j, and $d_{i}$ and $d_{j}$ are the number of fungal hosts associated with MH plant i and j, respectively. Note that the Bray–Curtis measure corresponds to the number of shared fungal hosts relative to the total number of fungal hosts. The second measure we used is the overlap measure $C_{ij}/\text{min}\left(d_{i},d_{j}\right)$, where the parameters are the same as above and $\text{min}(d_{i},d_{j})$ refers to the smallest of the two values (Saavedra, Rohr, Dakos, & Bascompte, 2013). The overlap measure corresponds to the number of shared fungal hosts relative to the maximum number of fungal hosts that can be shared. Correlations were computed using the function *mantel* in the *R‐vegan* package. Mantel statistics were tested for significance by permutation (10^4^ trials). Separately, phylogenetic signal on the shared fungal hosts was investigated among MH plants species that belong to the same location.

For each MH plant, the observed fungal‐host diversity was calculated using the phylogenetic diversity (PD) of the observed hosts. Phylogenetic diversity was calculated by summing up the branch lengths in the fungal‐host phylogenetic tree among all the fungal hosts associated with the MH plant or group. Because the number of fungal hosts determines the branch length, we normalized the PD by calculating the scaled PD as $PD^{\prime }=\left(PD-PD_{\min}\right)/\left(PD_{\max}-PD_{\min}\right)$, where $PD_{\max}$ and $PD_{\min}$ correspond, respectively, to the maximum and minimum PD values that can be generated from all the possible combinations of fungal hosts. These combinations are generated by creating groups of fungal hosts of the same number as in the observed case, but the identity of the hosts is changed using the pool of the 138 possible fungi. The MH plants from our study were only found to associate with these 138 fungi, which represent a subset of the total fungal diversity available in the soil. Note that this scaling does not assume a particular generative process, rather it compares the observed phylogenetic diversity to all the possible outcomes with the same number of fungal hosts.

### 2.3 Fungal‐host diversity and overlap among mycoheterotrophic plants

We investigated the diversity and overlap patterns among observed co‐occurring MH plants in the field, as well as among the artificially generated groups. In particular, we observed six communities of MH plants that were found to be co‐occurring in the field. To maximize the possibility of co‐occurrence and to avoid small‐scale niche segregation of mycorrhizal communities (Jacquemyn et al., 2014), plants were considered to co‐occur when flowering specimens were found to be growing less than one meter from each other (see Table S4 for the composition of these communities). Two of the observed communities in the field had two plants, three communities had three plants, and one community had five plants. Additionally, to generate groups of potentially co‐occurring plants, we formed all groups with n plant species using the 20 MH collected species. We generated artificial groups with two, three, four, and five MH species (mimicking the size of the observed communities in the field).

In every single observed community and generated group, we calculated the combined phylogenetic diversity (PD) of the fungal hosts that can be associated with a given community/group of MH plants. Similarly, to investigate fungal‐host overlap among MH plants, we calculated the overlap of fungal hosts among MH plants in a given community/group. This overlap is again calculated as $\sum_{i<j}C_{ij}/\text{min}\left(d_{i},d_{j}\right)$, where $C_{ij}$ represents the number of fungal hosts shared between MH plant i and j that belong to a given community/group, $\text{min}(d_{i},d_{j})$ refers to the smallest of the two values, and the summation is done over all possible pairs of MH plants (Saavedra et al., 2013). Note that this overlap measure corresponds to the average number of shared fungal hosts among all pairs of MH plants in given community/group relative to the maximum number of fungal hosts that can be shared. To compare phylogenetic diversity and overlap across communities/groups, we used the scaled PD and scaled overlap, which are the values of the phylogenetic diversity and overlap measures within the range of possible phylogenetic diversity and overlap values generated by all the groups with the same number of plants.

Finally, to investigate the spatial influence of our sampling in the observed patterns of fungal hosts in MH plants, we compared the scaled PD and scaled overlap between MH plants belonging to the same location and MH plants belonging to different locations. Because in nine of the fifteen localities we visited, we found more than one MH plant species (see Fig. S1), we generated two categories for each of the groups with two, three, four, and five plant species generated above. Only if all plants in a given group were found in a common location, they were considered in category one. Otherwise, the group was considered in category two. For each group and category, we separately calculated the scaled PD and scaled overlap.

## 3 Results

### 3.1 Fungal‐host diversity in single mycoheterotrophic plants

**Figure 2:**
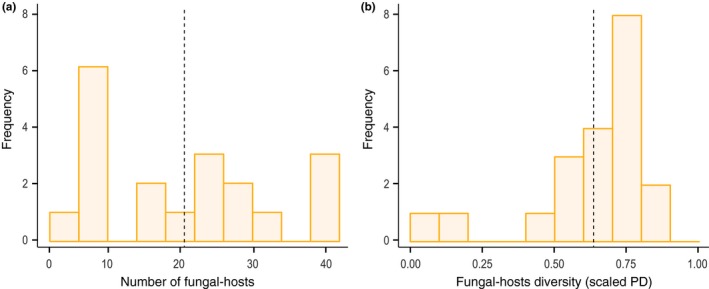
Fungal‐host patterns in single mycoheterotrophic plants. Panel (a) shows the distribution of the total number of fungal hosts associated with each of the 20 observed MH plants. Panel (b) shows the fungal‐host diversity (scaled phylogenetic diversity) associated with each of the 20 observed plants. This shows that most of the observed MH plants have a fungal‐host diversity that falls in the upper half of the potential range. The dashed lines correspond to the mean values in the distributions

We found that the number of fungal hosts in each of the 20 MH plant species varies from 2 to 42 (see Figure 2a). Particularly, we found no phylogenetic signal on the number of fungal hosts among plants (Mantel test: r=−.050, p=.766, df=19) nor on the fungal hosts shared among plants (Mantel tests: Bray–Curtis r=−.035, p=.682; overlap r=.047, p=.245; df=19). Looking at the MH plants that belong to the same location (Fig. S1), we found no phylogenetic signal on the number of fungal hosts among plants (Mantel test: r=.17, p=.375, df=3 for Laussat; r=−.20, p=.650, df=4 for Elie; r=−.21, p=.717, df=5 for Singes; r=.37, p=.089, df=5 for Virginie) nor on the fungal hosts shared among plants (Mantel test: Bray–Curtis r=.03, p=.583; overlap r=.03, p=.512; df=3 for Laussat; Bray–Curtis r=−.54, p=.983; overlap r=.34, p=.150; df=4 for Elie; Bray–Curtis r=−.22, p=.794; overlap r=.08, p=.472; df=5 for Singes; Bray–Curtis r=−.09, p=.608; overlap r=.25, p=0.161; df=5 for Virginie). Overall, these findings reveal an important variability in MH interactions that can be driven by mechanisms other than evolutionary relationships.

Additionally, we found that fungal‐host diversity in each observed plant ranks among the highest when compared to the potential host diversity that can be expected by chance in a single MH plant with the same number of fungal hosts. The majority of plants (14 of 20) lie in the upper half of the range of possible phylogenetic diversity values (scaled PD>0.5; Figure 2). These findings imply that individual plants typically have a high fungal‐host diversity by exploiting distantly related fungi, regardless of their number. This raises then the question of how plants are sharing their fungal hosts.

### 3.2 Fungal‐host diversity and overlap among mycoheterotrophic plants

Mycorrhizal fungi create extensive underground networks that could make MH plants compete to obtain their belowground vital resources via their MH interactions. This makes necessary the study of how the diversity of MH interactions is modulated and shared within groups of plants.

**Figure 3:**
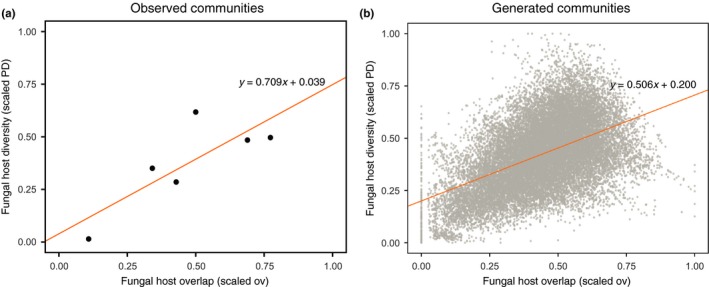
Fungal‐host diversity increases along with fungal‐host overlap among mycoheterotrophic plants. The figures show the relationship between fungal‐host diversity and fungal‐host overlap for both the six observed communities in the field (panel a) and in the artificially generated groups of plants (of the 20 sampled MH species) (panel b). Both panels show the common positive relationship between fungal‐host diversity (scaled phylogenetic diversity in *y*‐axis) and fungal‐host overlap (scaled overlap in *x*‐axis). Fungal‐host diversity and overlap correspond, respectively, to the combined phylogenetic diversity of the hosts associated with the plants in each group normalized by the number of fungal hosts, and the fraction of shared fungal hosts (see Section 2). The solid lines correspond to the linear regression between scaled PD and scaled overlap across all points

We find that on average the fungal‐host diversity (the combined phylogenetic diversity of the associated fungal hosts within the group) is proportional to fungal‐host overlap (the average fraction of shared fungal hosts) in groups of MH plants. This pattern was present in both the observed communities in the field (Figure 3a) and in the generated group of plants (Figure 3b). In particular, there is a systematic positive association between scaled PD and scaled overlap in the observed communities (Pearson's correlation: r=.805, p=.053, df=4) and in the artificially generated groups (Pearson's correlation: r=.497, p=.001, *df* = 21,680). This positive relationship does not depend on group size (Pearson's correlation: r=.377, df=191, p=.001 for two species, r=.487, *df *= 1,138, p=.001 for three species, r=.493, *df* = 4,843, p=.001 for four species, r=.478, *df* = 15,502, p=.001 for five species).

The results above are also qualitatively the same if scaled PD and scaled overlap values are replaced by their raw values while controlling for the total number of fungal hosts. Because the number of specimens and the OTU richness per MH species are variable among samples and may influence the results (see Table S1), we computed the partial Pearson's correlations between scaled PD and scaled overlap controlling for the number of individuals sampled per species, number of OTUs, and variation in the number of individuals per species within a community (using the Herfindahl index). The obtained correlations remain positive and significant at the 95% confidence, which confirm that fungal‐host diversity within a group of plants increases together with their fungal‐host overlap.

Finally, by dividing the categories of MH plants into one in which all plants belong to the same location and another one in which not all plants belong to the same location (see Section 2), we found that typically the former group displays higher levels of both scaled PD and scaled overlap across the different group sizes (see Tables 1 and 2). These results suggest that fungal‐host diversity increases within a location as a response to a natural increase in fungal‐host overlap, which can be expected from a niche framework perspective (Levine & HilleRisLambers, 2009; MacArthur & Levins, 1967; Rohr et al., 2016).

**Table 1:** Fungal‐host diversity is higher in groups of plants that belong to the same location. The table shows the *t*‐test results comparing the scaled PD in groups of MH plants (composed by two, three, four, or five species) that belong to the same location and in different locations

Scaled PD	Mean in same location	Mean in different location	*p*‐value	95% CI
Two species	0.421	0.297	.0012	0.05, 0.20
Three species	0.412	0.327	.0002	0.04, 0.13
Four species	0.479	0.394	.0009	0.04, 0.39
Five species	0.553	0.440	.0023	0.05, 0.18

**Table 2:** Fungal‐host overlap is higher in groups of plants that belong to the same location. The table shows the t‐test results comparing the scaled overlap in groups of MH plants (composed by two, three, four, or five species) that belong to the same location and in different locations

Scaled overlap	Mean in same location	Mean in different location	*p*‐value	95% CI
Two species	0.358	0.220	6.6 e‐6	0.07, 0.21
Three species	0.493	0.362	3.2 e‐8	0.09, 0.17
Four species	0.512	0.404	2.1 e‐8	0.08, 0.14
Five species	0.577	0.458	1.3 e‐5	0.08, 0.15

## 4 Discussion

Previous studies have investigated fungal‐host diversity of MH plants in relation to the fungal diversity associated with the surrounding green plants (Bidartondo, Bruns, Michael, Sérgio, & Read, 2003; Bidartondo et al., 2002; Bougoure, Ludwig, Brundrett, & Grierson, 2009; Cullings, Szaro, & Bruns, 1996; Roy, Whatthana, Richard, Vessabutr, & Selosse, 2009; Yamato et al., 2011). However, several MH species present vast geographic distributions despite being locally rare. Therefore, these surrounding plants may not be the exclusive factors determining fungal‐host diversity in MH plants. Indeed, many studies have reported the occurrence of different species of arbuscular mycoheterotrophs in the field without a clear explanation for this phenomenon (e.g. Cheek & Williams, 1999; Jonker, 1938; Maas & Rübsamen, 1986; van de Meerendonk, 1984; Merckx, 2013; van der Pijl, 1934; van Royen, 1972).

In our study, we have considered potential neighboring effects of MH plants with each other as possible drivers of fungal‐host diversity. Because many unmeasured factors can influence MH interactions, we opted to compare the observed patterns against all the possible fungal‐host combinations (what we called artificially generated groups of plants). We have found that individual MH plants have a tendency to exploit more distantly related fungi than expected by chance. This tendency of targeting distantly related fungi has been described in autotrophic plants (Giovannetti, Sbrana, Avio, & Strani, 2004). Nevertheless, it has been suggested that MH plants have more restricted interactions, as they often show higher specificity toward their fungal hosts (e.g. Bidartondo et al. 2002; Gomes, Aguirre‐Gutiérrez, Bidartondo, and Merckx 2017). For example, in *Afrothismia*, five closely related MH plants were found to specialize in five closely related lineages of Glomeromycota fungi (Merckx & Bidartondo, 2008). In contrast, in Monotropoideae, the five MH species in this clade associate with five different distantly related Basidiomycota fungi, but each within the same fungal lineage (Bidartondo & Bruns, 2005). Either way, and despite the processes leading to this extreme level of fungal specificity, it has been suggested that MH plants adapt to the suitable fungal partners that participate in this mycoheterotrophic interaction, and therefore, host‐jumps to distantly related fungal lineages are unexpected (Bidartondo & Bruns, 2002).

Building on niche theory, our results may reflect a MH plant strategy to increase its fungal‐host diversity or niche width, as species with a wider niche may be more likely to obtain different resources and to establish successfully in new habitats (Levine & HilleRisLambers, 2009; Levins, 1968; Tilman, Wedin, & Knops, 1996). Mycoheterotrophic plants require established mycorrhizal networks to persist (van der Heijden, Martin, Selosse, & Sanders, 2015; Sachs & Simms, 2006). Although each species tend to increase the phylogenetic diversity of their fungal hosts, it is still a limited fraction of the total diversity of arbuscular mycorrhizal fungi that can be part of this interaction (Douglas, 2008; Gomes et al., 2017; Merckx et al., 2009), suggesting that these fungi appear to be under selection pressure to be resistant to these cheaters (Douglas, 2008). Therefore, the ability to increase its fungal‐host diversity may confer an advantage to increase the opportunities to cheat mycorrhizal networks.

We have found that in communities of co‐occurring MH plant species in the field the fungal‐host diversity among MH plants appear to increase proportionally to their fungal‐host overlap. This same tendency was confirmed among the artificially generated groups of MH plants showing that the patterns observed are not an artifact of the reduced number of MH communities observed in the field. Moreover, we have found that both fungal‐host diversity and overlap are significantly higher among plants that belong to the same geographic location, which could provide an explanation for the lack of phylogenetic signal on the fungal hosts among MH plants. These results indicate that fungus‐plant interactions can be better explained by understanding plant–plant interactions generated by sharing resources or fungal hosts. Future studies could explain whether this symmetry between fungal‐host diversity and overlap may respond to an ecological mechanism driven by maximizing co‐occurrence and avoiding competitive exclusion among MH plants.

A potential bias in our study is the use of ITS2 sequences and future work should consider expanding these sequences (see Supporting Information for more details). Another aspect that deserves particular attention is the influence of abiotic factors that can affect the diversity of fungal hosts for the MH plants. In fact, many other factors can influence diversity, including the surrounding autotrophic plants. Taking everything into account is virtually impossible. However, our findings suggest that species coexistence cannot be fully understood without attention to the two sides of ecological interactions.

## Conflict of interest

The authors declare no competing financial interests.

## Data accessibility

Data are publicly available as Supporting Information.

## Author contributions

SS designed the overall study. VSFTM designed the sampling and DNA study and provided data. SIFG performed the analysis. SIFG and SS wrote a first draft of the manuscript; all authors contributed to revisions.
