# Supplementary material for: Fungal‐host diversity among mycoheterotrophic plants increases proportionally to their fungal‐host overlap
Source: Ecol Evol. 2017 Apr 22;7(10):3623–30. doi: 10.1002/ece3.2974 (PMC5433980; doi:10.1002/ece3.2974)
Supplement: Supplementary file 1 [file ECE3-7-3623-s001.docx]

**SUPPORTING INFORMATION**

**DNA sequencing:** Fungal DNA was extracted from root material with KingFisher Flex Magnetic Particle Processors (Thermo Scientific, USA), using the NucleoMag 96 Plant Kit (Machery-Nagel Gmbh and Co., Düren, Germany). The internal transcribed spacer 2 (ITS2) was amplified using the fungal specific primer flITS7 (Ihrmark et al., 2012) and the universal primer ITS4 (White et al., 1990), which was labeled with 96 different Ion Torrent MID-labels to differentiate individual samples. ITS2 labelled amplicons were sequenced on a Personal Genome Machine with 850 flows (PGM; Life Technologies, Guilford, CT, USA). The next-generation sequencing of the 140 specimens were done in two runs, including other plant species not used for this work. The reads obtained then were processed using USearch v.7 using the UPARSE algorithm (Edgar, 2013). The Ion Torrent runs originated 9,547,370 raw sequences. From these, 156,517 passed our quality control steps (excluding sequences with Q < 20, length < 100 bp and global singletons), originating 37,563 unique sequences. These sequences were clustered at 97% similarity. A chimera check was performed using Uchime Reference Database (3.07.2014 UNITE/INSD; (Edgar et al., 2011). Global OTUs singletons and doubletons were excluded, generating a total of 138 Glomeromycota OTUs (represented by 7,227 sequences). The 138 OTUs were identified by BLAST search using the UNITE+INSD database (version 6.0, 10.09.2014) in UPARSE implemented with the current Index Fungorum classification. See Table S2 in Supporting Information for information on the closest match for each OTU. We matched the 138 fungal hosts to the 20 MH plant species (see Table S3 in Supporting Information for presence/absence table). All non-Glomeromycota OTUs were omitted, retaining 138 Glomeromycota OTUs for further analysis. Because the majority of the Glomeromycota hits (see Table S2) matched uncultured Glomeromycota species, we placed the obtained OTUs in a phylogenetic tree (see Figure S3) to better understand their phylogenetic relationships.

To avoid the conflicts that molecular assessments generate in the species delimitation of arbuscular mycorrhizal fungi, due to the current absence of species concept for the fungi in this phylum, we measured the diversity of MH interactions as the phylogenetic diversity among the fungi detected per plant species, instead of considering the number of OTUs.

A potential bias in our study is the use of ITS2 sequences. The marker regions often used for Glomeromycota phylogenetic studies are ribosomal DNA markers, including SSU, ITS and LSU genes, also because rDNA markers are the largest sampled within this group of fungi. Previous studies showed that SSU alone has a limited resolution power (Bruns et al., 1991; Hofstetter et al., 2007), which can introduce a bias towards an under-estimation of AM fungi (Krüger, 2011). The ITS region is known to be a highly variable region, which can also introduce a bias in the opposite direction of the SSU marker, towards an over-estimation of AM fungi. To overcome these problems, Krüger et al. (2009) suggested the amplification of a SSU-ITS-LSU fragment for a phylogenetic analysis with species-level resolution. However, the use of next-generation DNA sequencing techniques only allows amplification of short DNA fragments, which forces us to choose a fragment of one of the three markers. Due to the limited length of Ion Torrent sequencing, the better candidate region chosen was the ITS2. Preliminary data analysis (not shown) based on the SSU region has proven not to discriminate the different fungal lineages associated with these plants. Therefore, the use of ITS2, which is a more variable region, potentially delivers the most phylogenetic informative characters. Because ITS2 is a fast-evolving region we used a backbone alignment including concatenated reference sequences (Krüger et al., 2012) of partial SSU, whole ITS and partial LSU representative of all the described AM fungi genera (Krüger et al., 2009), adding the two new genera described later on curated in the MaarjAM database (Öpik et al., 2010), for a more accurate phylogenetic placement of the generated fungal sequences in this study. To reduce the potential bias due to the under- or over-splitting of fungal taxa in OTUs, we used the phylogenetic distances between the fungal taxa instead of richness of the samples in the downstream analysis.

**Fig. S1:** Map of the 15 sampling locations of our study: 10 in French Guiana and 5 in

Brazil. Mycoheterotrophic species (on the left) are represented by numbers in each location

that were collected (on the right).

**Fig. S2:** Phylogeny of MH plants used to infer phylogenetic signal. Branch lengths represent

divergence times. Root age and crown node ages of the sampled families are shown (in

million years ago).





**Fig. S3:** Phylogeny of the Glomeromycota OTUs found in all the MH plants. Sequences

with identification correspond to curated sequences of Glomeromycota (see DNA Sequencing

in Supporting Information). Sequences indicated with cons were obtained from the

reference dataset of AM fungi built by Krüger et al. (2011). We also included the following

genera identified in the MaarjAM database (Öpik et al., 2010): Dominikia spp.

(HG938301-HG938304, KJ564145-KJ564169, KM05657-KM05665, KR105638-KR105649),

Kamienskia spp. (KJ564133-KJ564144), Redeckera spp. (HG518627-HG518629), Septoglomus

spp. (HF548853-HF548862). The list of OTU numbers in the collapsed clades is the

following OTUs A: 7, 36, 41, 42, 56, 65, 79, 82, 97, 100, 136, 170, 203, 210, 225, 293, 325,

438, 471, 497, 499, 508, 545, 641, 683, 765, 777, 798, 819, 956, 1048, 1109, 1255, 1257,

1353, 1355, 1362, 1594, 1939, 2129, 2182, 2186, 2191, 2239, 2266, 2443, 2509, 2518, 2586,

2660, 2847, 2898, 3037, 3062, 3094, 3239, 3386, 3515, 3581, 3700; OTUs B: 338, 873, 1377,

1625, 2613; OTUs C: 12, 45, 57, 80, 81, 159, 162, 211, 233, 253, 260, 320, 354, 364, 382,

506, 523, 553, 653, 686, 687, 769, 772, 997, 1002, 1034, 1052, 1064, 1079, 1086, 1135, 1357,

1381, 1492, 1512, 1633, 1664, 1788, 2072, 2198, 2304, 2312, 2319, 2402, 2424, 2772, 3171,

4185, 4203; OTUs D: 400, 590; OTUs E: 38, 299, 1349, 1656, 2112, 3260, 3355; OTUs F:

107, 112, 146, 383, 786, 1642, 1677.

**
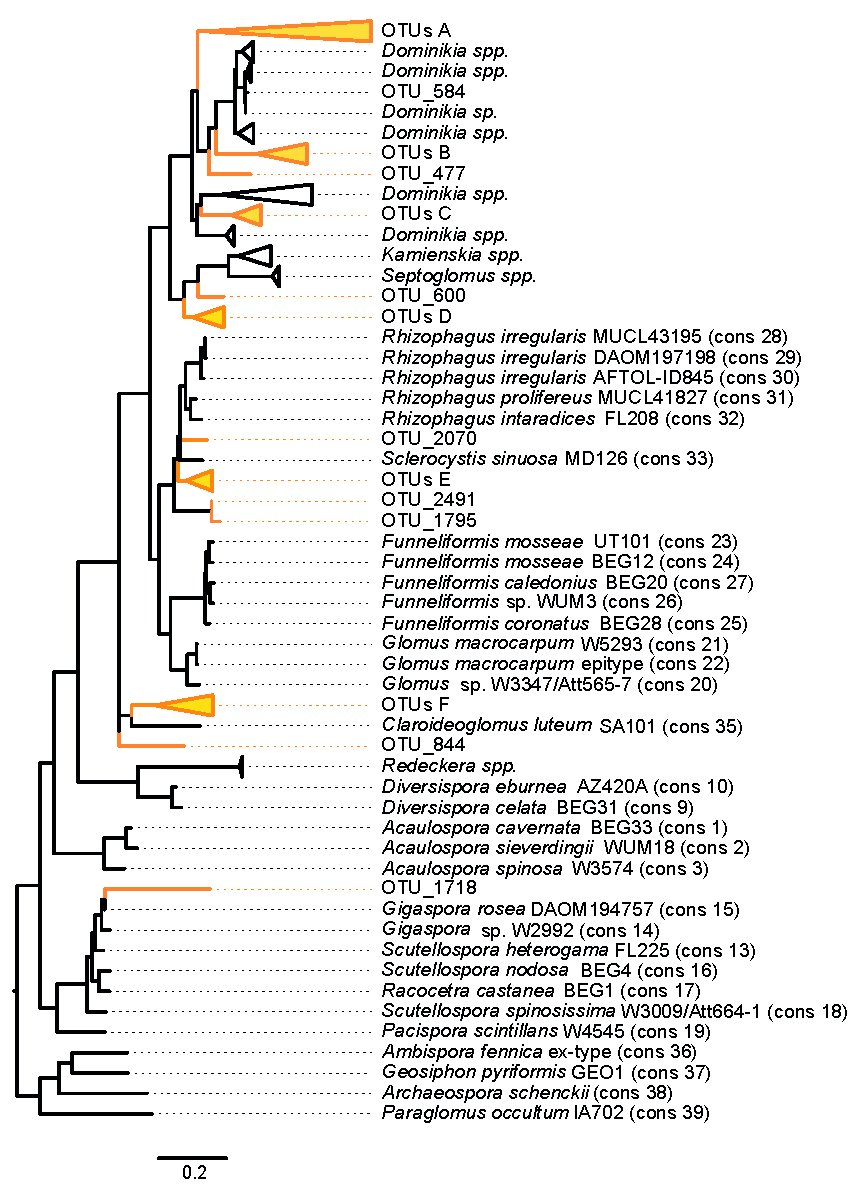
**

**Table S1:** Identity of MH plant species. Detailed sample localities with GPS coordinates

and collection dates are presented for each sampled specimen. Specimens coded with the

same collection number followed by underscore and specimen number were collected less

than 1 m apart from each other; different collection numbers indicate that specimens were

collected isolated. It is represented the number of reads generated by next generation

sequencing (after filtering steps), OTUs detected per plant specimen and total unique

OTUs per plant species.

**Table S2:** BLAST hits for the Glomeromycota OTUs based on the UNITE database. For

each OTU, the closest match is presented.

**Table S3:** Overview of the number of OTUs and number of sequences generated per sample.

Presence of each OTU is shown per sample.

**Table S4:** Species composition of the co-occurring MH plant communities. Plants were considered to co-occur when flowering specimens were found growing less than 1 meter apart from each other. We observed six communities of co-occurring species.


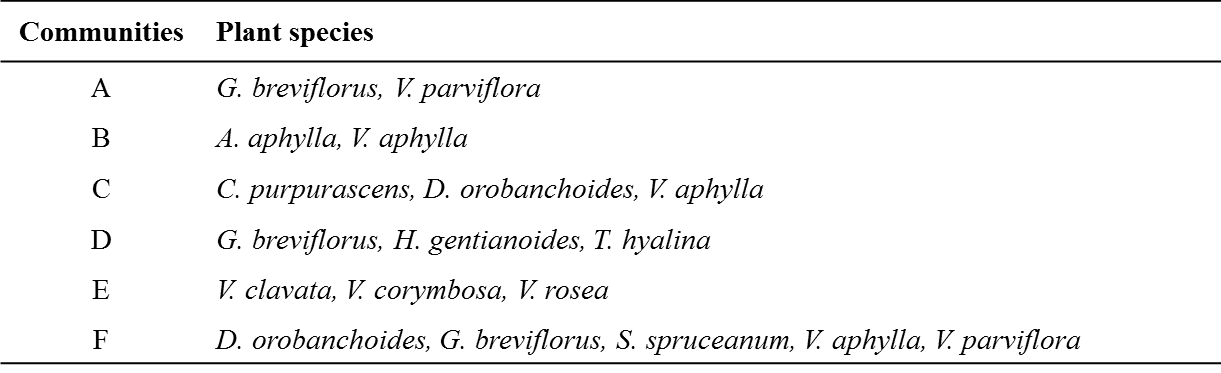


**Additional references:**

Bruns, T., White, T., & Taylor, J. (1991). Fungal molecular systematics. *Annual Review of Ecology and Systematics*, 22, 525–564.

Edgar, R. C. (2013). UPARSE: Highly accurate OTU sequences from microbial amplicon reads. *Nature Methods*, 10, 996–998.

Edgar, R. C., Haas, B. J., Clemente, J. C., Quince, C., & Knight, R. (2011). UCHIME improves sensitivity and speed of chimera detection. *Bioinformatics*, 27, 2194–2200.

Hofstetter, V., Miadlikowska, J., Kau, F., & Lutzoni, F. (2007). Phylogenetic comparison of protein-coding versus ribosomal RNA-coding sequence data: A case study of the Lecanoromycetes (Ascomycota). *Molecular Phylogenetics and Evolution*, 44, 412–426.

Krüger, M. (2011). PhD thesis: Molecular phylogeny, taxonomy and evolution of arbuscular mycorrhizal fungi DNA-based characterization and identification of Glomeromycota. Ludwig-Maximilians University Munich.

Krüger, M., Krüger, C., Walker, C., Stockinger, H., & Schüßler, A. (2012). Phylogenetic reference data for systematics and phylotaxonomy of arbuscular mycorrhizal fungi from phylum to species level. *New Phytologist*, 193, 970–984.

Krüger, M., Stockinger, H., Krüger, C., & Schüßler, A. (2009). DNA-based species level detection of Glomeromycota: One PCR primer set for all arbuscular mycorrhizal fungi. *New Phytologist*, 183, 212–223.
